# Review 3D-Printed hydrogels for tissue engineering: a review

**DOI:** 10.3389/fbioe.2026.1851399

**Published:** 2026-06-10

**Authors:** Chao-Ming Su, Jian-Jr Lee, Ming-You Shie, Hong-Kai Chen, Yu-Cheng Chang, Febrian Ruciyanti, Lathifa Diyang Wibawa, Faradilla, Muhammad Aulia Alfarisi, Yi-Wen Chen, Yu-Fang Shen

**Affiliations:** 1 Advanced Therapeutic and Pharmaceutical Center, China Medical University Hospital, Taichung, Taiwan; 2 School of Medicine, China Medical University, Taichung, Taiwan; 3 Department of Plastic and Reconstruction Surgery, China Medical University Hospital, Taichung, Taiwan; 4 Xenotransplantation Translational Research Center, China Medical University Hospital, Taichung, Taiwan; 5 School of Dentistry, China Medical University, Taichung, Taiwan; 6 Department of Bioinformatics and Medical Engineering, Asia University, Taichung, Taiwan; 7 Department of Cardiology, Asia University Hospital, Taichung, Taiwan; 8 Graduate Institute of Biomedical Sciences, China Medical University, Taichung, Taiwan; 9 High Performance Materials Institute for xD Printing, Asia University, Taichung, Taiwan

**Keywords:** 3D printing, digital light projection (DLP), natural hydrogels, printable hydrogels, stereolithography (SLA), synthetic hydrogels, tissue engineering, tough hydrogels

## Abstract

Three-dimensional (3D) bioprinting of hydrogels has emerged as an important strategy in tissue engineering because it enables the fabrication of scaffolds with controllable architectures, tunable properties, and biomimetic microenvironments. This review primarily focuses on recent advances in hydrogel-based 3D bioprinting over the past 2 decades, while also incorporating selected landmark studies to provide historical context for the evolution of the field. Major hydrogel printing techniques, including light-based, extrusion-based, and inkjet-based systems, are discussed and compared in terms of printing resolution, structural fidelity, material compatibility, cytocompatibility, and practical limitations. In addition, the roles of natural and synthetic hydrogels are examined, highlighting their distinct yet complementary advantages in bioactivity, printability, and mechanical performance. Recent progress in tough and composite hydrogels is further reviewed, with emphasis on strategies such as nanocomposite reinforcement, supramolecular interactions, double-network formation, gradient structures, and hydrogel–polymer hybrids for improving the mechanical durability and functional performance of printed constructs. The review also summarizes emerging applications in wound healing, cartilage repair, bone and osteochondral engineering, vascularized tissue fabrication, and other tissue-specific systems, while addressing major translational challenges, including scalability, vascularization, reproducibility, manufacturing standardization, and regulatory considerations. Overall, this review provides an updated and integrated perspective on the design, fabrication, functional optimization, and clinical translation potential of 3D-printed hydrogels for tissue engineering applications.

## Graphical Abstract

**Figure:**
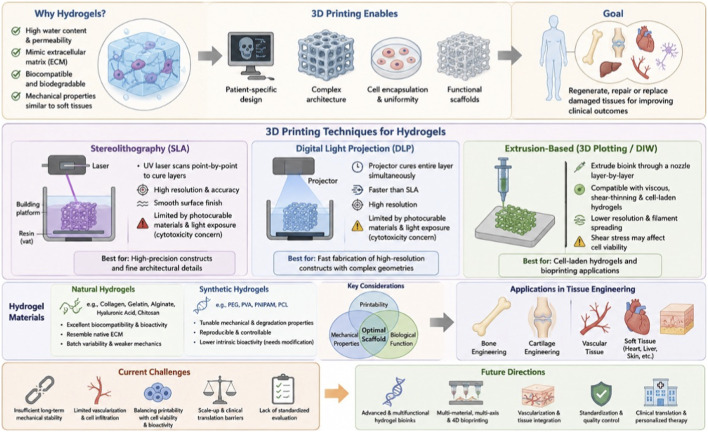

## 1 Introduction

The increasing gap between the demand for and supply of organs and tissues has made tissue engineering an important research field. The advent of biocompatible materials opened up the possibility of generating new tissues by placing living cells on appropriately designed scaffolds, and this breakthrough marked the beginning of the field of tissue engineering. Tissue engineering aims to create living substitutes for damaged tissues in the body (Drury and Mooney, 2003), which integrates principles from materials engineering and life sciences to regenerate, repair, replace, enhance and maintain tissues affected by disease, injury or congenital abnormalities (Advincula et al., 2021).

To create the scaffolds required for tissue engineering, various materials are utilized. Among the most commonly used materials for scaffolds are hydrogels. In the past 20 years, hydrogels have become increasingly popular as scaffolds for tissue engineering due to their ability to provide mechanical support for cells in engineered tissues, imitate the natural extracellular matrix, and maintain a unique 3D structure (El-Sherbiny and Yacoub, 2013).

Hydrogels are water-rich polymeric materials that maintain a three-dimensional network structure without dissolving in physiological environments. They are highly permeable to molecules, with diffusivity similar to that of liquids, rather than solids. For instance, oxygen, glucose, and albumin can diffuse through polyethylene glycol (PEG) hydrogels at rates of 100 μm^2^/s, 50 μm^2^/s, and 10 μm^2^/s, respectively, which is only slightly lower than their diffusivity in water. In contrast, hydrophobic materials like natural rubber have much lower diffusivity, with oxygen diffusing 1000 times slower in rubber than in water, while glucose and proteins show even lower diffusivity. Given their solid form and high permeability, hydrogels have found extensive use in medicine for the replacement of soft and hard tissues (Baroli, 2007).

In recent decades, hydrogels composed of natural biopolymers, such as hyaluronic acid, collagen, alginate, and chitosan, have gained significant attention in tissue repair applications (Cao et al., 2021). These hydrogels are favored due to their superior biocompatibility and non-toxic degradation byproducts. However, synthetic hydrogels face challenges in tissue replacement due to our limited understanding of the fate and toxicity of their degradation by-products after implantation, and the impact of engineered hydrogels on cells. Therefore, it is crucial to develop novel hydrogels with controllable degradation and non-toxic degradation products that can support implanted cells’ function and maturation to a specified lineage and phenotype (Li et al., 2018).

Recent advances in tissue engineering have made 3D bioprinting an attractive approach to scaffold creation. Bioprinting is a cutting-edge technology in the field of medical science that uses computer-assisted methods to create layered structures of live cells and biological materials. With the use of biocompatible inks, this innovative approach has revolutionized the manufacturing process by replacing metal raw materials or traditional plastic. In 1988, Klebe first demonstrated bioprinting under the name of cytoscribing technology, which positioned live cells to create 2D synthetic tissues (Klebe, 1988). Bioprinting has since expanded to 3D for scaffold engineering, soft tissue engineering, tissue renewal, and other fields, and has opened up new applications in the health field, including experimental research on artificial organ printing, the completion of missing bone parts, and personal prosthetics build (Bozkurt and Karayel 2021). Additionally, the utilization of 3D bioprinters enables the creation of biomimetic 3D biological tissue with high precision using direct replicas of a patient’s anatomical dimensions obtained from various scanning systems including X-rays, magnetic resonance imaging (MRI) scans, and computed tomography (CT) scans to offer a straightforward and effective way to create complex 3D structures (Xie et al., 2020). This technology allows for the incorporation of intricate geometric shapes and uniform distribution of live-cell types, biochemical structures, and biological materials, making it a revolutionary approach to tissue engineering. These characteristics make bioprinting a promising platform for the fabrication of functional tissue constructs (Topuz et al., 2018).

**FIGURE 1:**
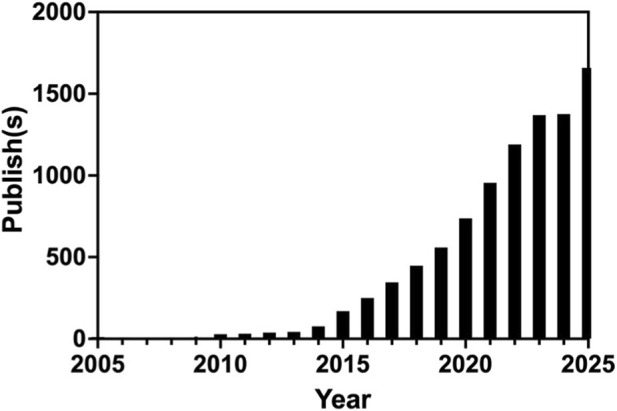
Annual publication trend of 3D bioprinting research based on Web of Science records.

As shown in Figure 1, the annual number of publications related to 3D bioprinting has increased dramatically over the past decade based on Web of Science statistics. Before 2014, the number of publications remained very limited, indicating that 3D bioprinting was still at an early stage of development. However, publication output began to rise steadily after 2015 and accelerated markedly after 2018, reflecting growing interest in biofabrication, hydrogel-based bioinks, and tissue-specific scaffold design. By 2025, the annual number of publications had exceeded 1,500, demonstrating that 3D bioprinting has evolved from an emerging concept into a rapidly expanding research field. This strong upward trend also suggests increasing recognition of its potential in tissue engineering, regenerative medicine, and personalized biomedical applications.

This review primarily focuses on the development of hydrogel-based 3D bioprinting over the past 2 decades, with particular emphasis on recent advances in printing technologies, hydrogel materials, and tissue-engineering applications. Earlier landmark studies are also included where necessary to provide historical context for the evolution of the field. The cited literature was selected to provide both historical background and a focused overview of recent technological and biomedical progress in this area.

## 2 3D printing techniques for hydrogel

### 2.1 Laser printing

#### 2.1.1 Stereolithography (SLA)

Stereolithography (SLA) is a light-based 3D printing technique that utilizes laser-induced photopolymerization. This technique is based on the photopolymerization of photocurable resins or hydrogel precursors, which is an exothermic polymerization process characterized by chemical cross-linking reaction using UV light (Bártolo, 2011). It will solidify photocurable liquid hydrogels or resin layer by layer. The SLA process consists of a vat filled with photocurable liquid resin (or in this case, hydrogels), a laser unit or laser source that directs an ultraviolet beam to a reflective mirror, a galvo motor system steers to move the mirror for focusing the beam to the resin surface, and a three-axis system to control the movement of the horizontal (X and Y-axis) and the vertical (Z-axis) platform which controlled by programmed system based on the STL file added before. Shows the illustration of the stereolithography system to make a 3D printing model (Huang J. et al., 2020).

Stereolithography 3D printing provides numerous benefits, including a large building volume that can vary from 200 to 2000 mm, exceptional structural resolution, and superior surface finishes. Moreover, the accurate control of parameters like scanning speed, exposure time, UV laser power, spot size, and wavelength can lead to the production of highly precise and uniform 3D printed objects (Chua and Leong, 2014).

**FIGURE 2:**
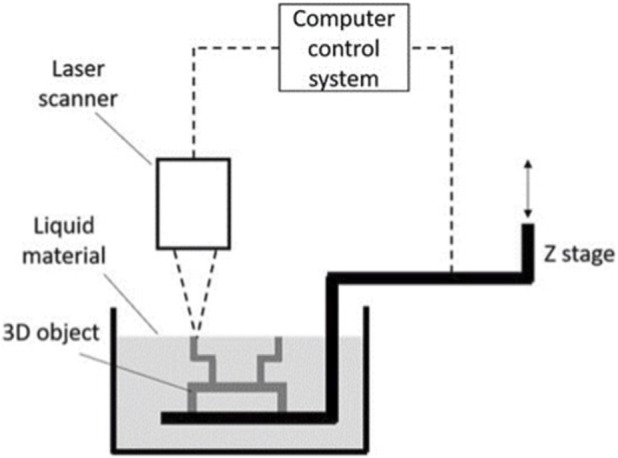
Schematic illustration of the stereolithography (SLA) system. A focused ultraviolet laser beam is directed onto a vat of photocurable resin or hydrogel precursor and scans the material surface in a layer-by-layer manner. Polymerization occurs at the irradiated regions, enabling the fabrication of three-dimensional structures with high precision under computer-controlled platform movement (Huang J. et al., 2020).

As illustrated in Figure 2, the SLA system is based on point-by-point photopolymerization of a liquid resin or hydrogel precursor under computer-controlled beam guidance. This configuration explains why SLA can achieve high structural precision and smooth surface finish, but also why its fabrication speed is relatively limited compared with projection-based methods such as DLP. Therefore, Figure 2 not only illustrates the device architecture, but also helps explain the characteristic trade-off of SLA between geometric accuracy and printing efficiency.

#### 2.1.2 Two-photon polymerization (TPP)

Without using the layer-by-layer method, two photon polymerization (TPP) is an advanced stereolithography-based technique for fabricating nano-resolution structures. The TPP system uses femtosecond infrared laser pulses to start the photolytic polymerization process without the usage of masks by focusing them into the volume of photocurable liquid materials (Huang Z. et al., 2020).

In comparison to UV light, infrared light may directly fabricate complex 3D structures with a significantly greater structural resolution of up to 200 nm due to its nonlinear behavior and the presence of polymerization threshold intensity. As a result, TPP-fabricated structures generally exhibit higher structural precision than those made using traditional SLA methods (Jang et al., 2018). Shows the illustration of TPP’s working principle.

To manage the scanning of an ultra-short laser pulse with an extremely small focusing spot size, a computer positioning system is used in combination with piezoelectric stages and/or optical scanning systems. This allows for precise control of the tightly concentrated beam. The intense photons from the two-focused beam source excite photo-initiator molecules, resulting in the production of free radicals. These free radicals initiate the polymerization process by breaking the unstable connection between monomers. This process creates and expands polymer chains by adding to existing chains and mixing monomers (O'Halloran et al., 2023). However, despite advances in photosensitive materials and initiators, there is still a problem with the lack of free radical density within the incredibly narrow cross-sectional focusing region for two photons.

**FIGURE 3:**
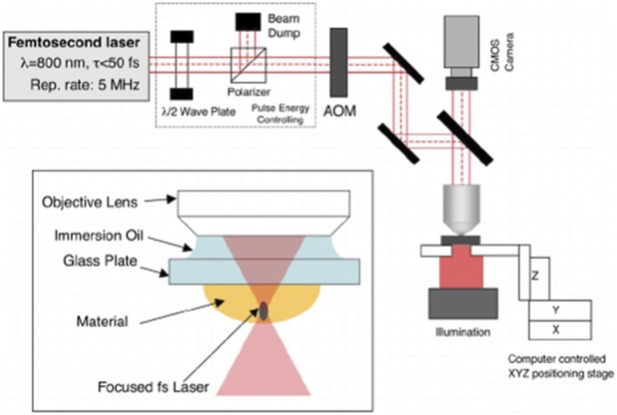
Schematic illustration of the two-photon polymerization (TPP) system. A femtosecond laser beam is directed through optical components and focused by an objective lens into the photosensitive material through a glass plate and immersion oil. Polymerization occurs only within the highly confined focal volume, enabling the fabrication of complex three-dimensional microstructures with extremely high spatial resolution. The system is integrated with a computer-controlled XYZ positioning stage and imaging module for precise patterning and monitoring (Paz et al., 2012).

Figure 3 highlights the highly localized polymerization region generated by a tightly focused femtosecond laser, which is the key reason why TPP can fabricate structures with micro-to nanoscale precision. At the same time, this figure also helps explain an important limitation of TPP, namely, that such extreme spatial control is achieved at the expense of throughput and scalability. Thus, the figure reinforces the idea that TPP is particularly valuable for highly complex microarchitectures, but less suitable for large-volume scaffold fabrication.

#### 2.1.3 Laser-induced forward transfer (LIFT)

Laser-induced forward transfer (LIFT) is a one-step direct laser writing process that involves three main components: a pulsed laser that is focused on a thin layer of laser-absorbing materials, such as metal or hydrogels, a donor substrate, which can be a glass print ribbon or laser-transparent quartz, and an acceptor or receiving substrate where the ink is deposited (Serra and Piqué, 2019).

In order to provide a transfer material source, a liquid, solid, or paste layer is applied to the laser-transparent donor slide. Once the laser pulses penetrate the donor slide and the coating layer absorbs the energy, the ink components are propelled towards the acceptor substrate and expelled from the coating layer once the incident energy reaches a pre-determined threshold (Delaporte et al., 2016).

#### 2.1.4 Digital light projection (DLP)

Digital light projection (DLP) is a 3D printing technique that shares similarities with stereolithography (SLA). The process includes various essential components, such as a digital light projector screen that acts as the light source for the DLP 3D printer, a Digital Micromirror Device (DMD) that controls the light beam emitted by the digital light projector, a vat containing hydrogels, a build plate for attaching the printed object, and a z-axis elevator responsible for raising the build plate (Ge et al., 2022).

Unlike SLA, DLP uses digital mirror devices or liquid crystal displays as the dynamic pattern-generating mask. Each pixel unit of the LCD or mirror is programmed to provide an individual on-off beam signal. Additionally, multi-lens components are used to concentrate the light beam sources, allowing the DLP light source to produce 3D structures with high resolution ranging from 25 to 150 microns. DLP operates on a top-down manufacturing concept, which differs from other 3D printing methods that use a bottom-up manufacturing approach (Jang et al., 2018).

### 2.2 Extrusion printing (nozzle-based)

Extrusion-based 3D printing is a method that utilizes a mobile nozzle to apply molten or semi-molten materials, such as polymers, polymer solutions, pastes, or dispersions, in a layer-by-layer fashion with computer-controlled precision. This approach is commonly used in direct ink writing mode. Extrusion printing can be divided into two main categories: melting-based processes, which include techniques like melt electrospinning writing and fused deposition modeling, and dissolution-based processes like 3D plotting (Lewis, 2006).

#### 2.2.1 3D plotting

The 3D plotting method involves the use of viscous hydrogels that are filled into a syringe and subsequently pushed out through a micro-needle into a liquid solution with a similar density to the hydrogel. This process results in the formation of a single, continuous micro-strand or micro-dots (Puertas-Bartolomé et al., 2020). The thickness of the product can be controlled by adjusting various factors such as the hydrogel viscosity, deposition rate, nozzle diameter, and applied pressure. The construction platform is usually stationary while the material dispensing head moves along the x, y, and z-axes. This movement can be achieved through the use of filtered air pressure (for pneumatic nozzles) or stepper motors (for volume-driven injection nozzles) to facilitate liquid flow. Matching the density of the hydrogel with the density of the liquid solution is crucial for the effectiveness of this technique (Jang et al., 2018).

#### 2.2.2 Direct ink writing (DIW)

Lewis et al. were the pioneers in investigating the use of direct ink writing (DIW) or direct write assembly (DWA) for 3D printing (Lewis, 2006). The DIW process is capable of printing a range of inks, including hydrogels, organic inks, colloidal suspensions, nanoparticle-filled inks, and gels. To conduct DIW printing, there are several essential components required, such as a cylindrical nozzle, a three-axis translation platform, pressurized air supply, and an optical microscope for real-time monitoring (Lewis, 2006).

**FIGURE 4:**
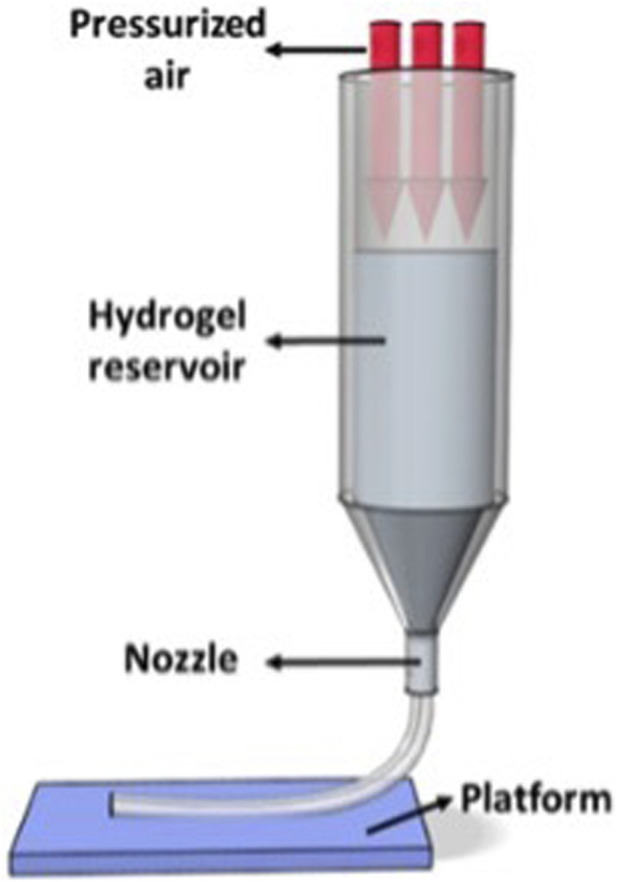
Schematic illustration of the direct ink writing (DIW) system. Hydrogel ink is continuously extruded through a nozzle under controlled pressure and deposited in a layer-by-layer manner onto the printing substrate. The system relies on appropriate rheological properties of the ink, such as shear-thinning behavior and shape retention, to maintain filament fidelity and structural stability during fabrication (Suntornnond et al., 2016).

When utilizing hydrogels for DIW printing, two important factors need to be considered. First, the hydrogels must have self-supporting capacity and regulated viscoelastic properties to maintain their shape during printing. Therefore, the hydrogels should exhibit rapid post-extrusion recovery to ensure that the printed structures retain their features. Second, a high nanoparticle or colloid concentration in the hydrogels is recommended to minimize shrinking during the drying of the final assembly. For building planar and spanning filaments, the ideal solid loadings in hydrogels typically range between 70 and 85 weight percent (Ahn et al., 2011). In hydrogels, the nanoparticle or colloid network can withstand the compression force brought on by capillary tension, which prevents spreading during extrusion. As shown in Figure 4, DIW relies on continuous extrusion of hydrogel ink through a nozzle under controlled pressure, making rheological behavior a central determinant of print quality. This schematic therefore helps clarify why shear-thinning properties, rapid recovery after extrusion, and adequate self-support are essential for maintaining filament fidelity and overall construct stability. In this sense, Figure 4 serves not only as a technical illustration of the system, but also as a visual summary of the close relationship between ink rheology and printability (Suntornnond et al., 2016).

#### 2.2.3 Low-temperature deposition and manufacturing (LDM)

**FIGURE 5:**
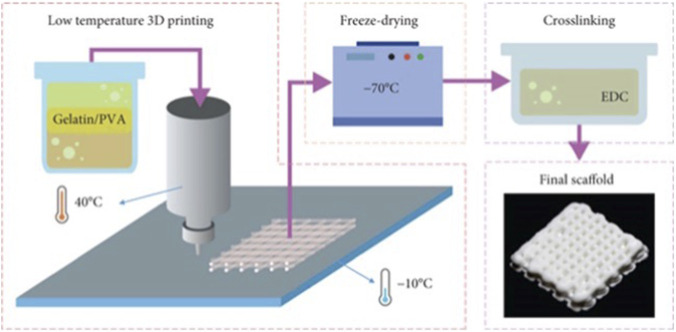
Schematic illustration of the low-temperature deposition manufacturing (LDM) system. Hydrogel or polymeric material is extruded onto a low-temperature platform, where the deposited strands are rapidly solidified under subzero conditions. This process supports temporary structural stabilization during printing and is commonly followed by freeze-drying to generate porous scaffold architectures (Liu et al., 2019; Kim et al., 2018).

The LDM process utilizes a lower temperature to solidify materials and involves a feeder that is connected to a screw pump nozzle for transferring substances like hydrogels onto a construction stage that is kept at a temperature below 0 °C. Subsequently, a freeze-drying technique is used to eliminate the solvent from the printed scaffold. Figure 5 illustrates that LDM combines extrusion with low-temperature solidification, which distinguishes it from conventional room-temperature extrusion methods. This feature is important because temporary freezing can improve structural retention during fabrication and facilitate the generation of porous scaffolds after freeze-drying. However, the figure also implies a practical limitation of this method, namely, that the requirement for subzero processing and post-print drying makes LDM less straightforward for direct cell-laden bioprinting. (Liu et al., 2019; Kim et al., 2018).

### 2.3 Inkjet printing

The inkjet printing process involves the projection of ink droplets onto a surface without the need for physical contact. This method employs a construction platform capable of vertical movement along the Z-axis, along with a printing head that can move horizontally in the XY plane and is equipped with a liquid binder cartridge (Singh et al., 2010).

#### 2.3.1 Inkjet-based 3D printer with powder (I3DP-P)

**FIGURE 6:**
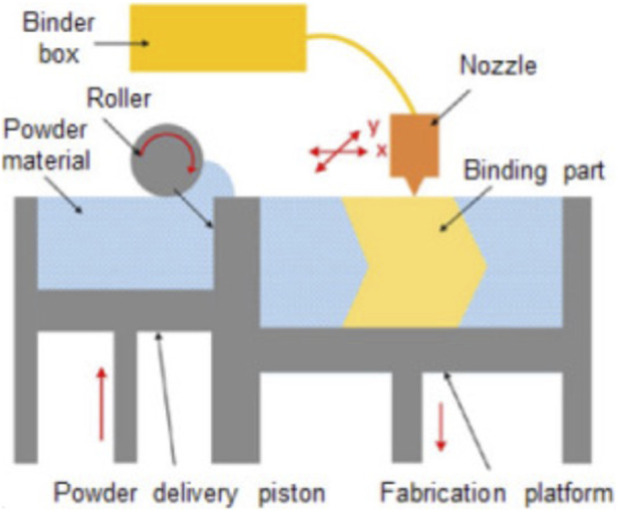
Schematic illustration of the inkjet-based 3D printing with powder (I3DP-P) system. A liquid binder is selectively deposited onto successive powder layers in a patterned manner, binding surrounding particles to form the desired three-dimensional structure. The surrounding unbound powder acts as a temporary support material during fabrication (Jang et al., 2018; He et al., 2016).

The I3DP-P technology is a rapid prototyping method that utilizes solid phase printing (Singh et al., 2010). It involves three primary steps in the process. Firstly, a platform is utilized to apply a layer of powder onto it. Secondly, a liquid binder is deposited onto the powder layer from an inkjet print head in a 2D pattern, sticking the nearby powder particles together. This process is repeated until the object is fully fabricated. One significant benefit of this method is that no supporting material is required since the binders in the unreacted powder can sustain the bound structures (Singh et al., 2010). The I3DP-P technique can use different types of powder, including single powder, surface-coated powder, or a combination of several powders. Choosing a suitable biocompatible powder and binder is a crucial aspect of the I3DP-P system. As illustrated in Figure 6, the powder-supported nature of I3DP-P reduces the need for additional supporting structures during fabrication, which is advantageous for maintaining the shape of the printed object. At the same time, this figure also reflects the dependence of the method on appropriate powder–binder combinations, which can restrict its direct use for hydrogel-rich or cell-laden bioprinting applications. Therefore, Figure 6 helps connect the operational principle of I3DP-P with both its fabrication advantages and its biological limitations (Jang et al., 2018; Liu et al., 2019; He et al., 2016).

#### 2.3.2 Inkjet-based 3D printer with liquid (I3DP-L)

**FIGURE 7:**
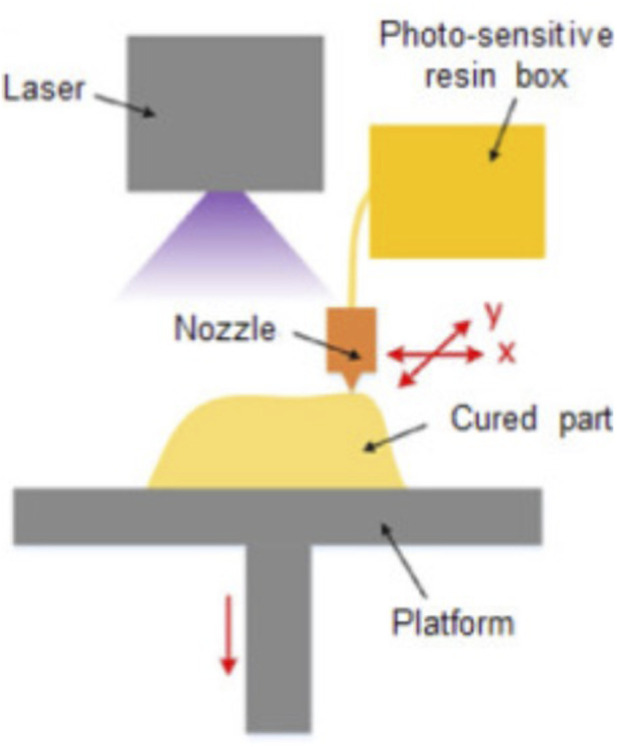
Schematic illustration of the inkjet-based 3D printing with liquid (I3DP-L) system. Liquid droplets of hydrogel precursor or crosslinking solution are precisely deposited onto a liquid-based substrate in a controlled pattern. Depending on the system design, crosslinking occurs through chemical or photo-induced processes, allowing accurate fabrication of hydrogel-based structures from low-viscosity printable formulations (Jang et al., 2018; Liu et al., 2019; He et al., 2016).

The I3DP-L technology consists of two types. The first type of system operates similarly to the I3DP-P technology but with a liquid bed instead of a powder bed (Jang et al., 2018). In this method, an uncross-linked hydrogel is filled into a bed platform that moves along the Z-axis, and a liquid crosslinker ink, such as calcium chloride, is deposited by the print head. The second type is a direct inkjet writing system, which usually utilizes photosensitive polymers. In this approach, photosensitive resin is discharged from inkjet printed heads, and simultaneous light curing occurs. Although the I3DP-L is more expensive than the I3DP-P, it has higher accuracy. Figure 7 shows that I3DP-L is based on precise liquid deposition and localized crosslinking, which explains its relatively high patterning accuracy compared with bulk extrusion methods. However, the figure also reflects an important limitation of the system, namely, that it generally requires low-viscosity printable formulations, thereby narrowing the range of compatible hydrogel materials. In this way, Figure 7 supports the broader comparison that higher accuracy in printing is often accompanied by more restrictive material requirements (Jang et al., 2018).

To facilitate a clearer technical comparison, the major 3D printing techniques used for hydrogel fabrication in tissue engineering are summarized in Table 1. These methods differ in their working principles, printable material requirements, resolution, printing speed, and suitability for biological applications. In general, light-based techniques such as SLA, TPP, and DLP offer high structural precision, whereas extrusion-based methods such as 3D plotting, DIW, and LDM provide broader material compatibility and are more suitable for viscous or cell-laden hydrogel systems. Inkjet-based approaches, in contrast, enable non-contact deposition and high accuracy but are typically limited by the low viscosity requirement of printable inks. Therefore, each printing technique presents distinct advantages and limitations, and the choice of method should be matched to the rheological properties of the hydrogel, the required structural fidelity, and the intended tissue-engineering application.

**TABLE 1:** Comparison of 3D printing techniques for hydrogels used in tissue engineering.

Printing technique	Working principle	Typical printable materials	Main advantages	Main limitations	Representative applications	Refs
Stereolithography (SLA)	UV laser scans photocurable resin/hydrogel layer by layer to induce crosslinking	Photocurable hydrogels, methacrylated polymers	High resolution, smooth surface finish, precise parameter control	Limited material selection, possible UV-induced cytotoxicity, slower point-by-point scanning	High-precision scaffold fabrication	Bártolo (2011), Huang et al. (2020a), Chua and Leong (2014)
Two-photon polymerization (TPP)	Focused femtosecond infrared laser induces localized two-photon polymerization in photosensitive materials	Photosensitive hydrogels/resins	Nano/microscale resolution, highly complex 3D microstructures	High cost, low throughput, limited scalability	Micro/nano tissue scaffolds, microdevices	Huang et al. (2020b), Jang et al. (2018), O'Halloran et al. (2023)
Laser-induced forward transfer (LIFT)	Laser pulse transfers material from donor slide to receiver substrate without nozzle contact	Liquid, paste, hydrogel inks	High resolution, nozzle-free deposition, suitable for cell printing	Complex setup, limited large-scale fabrication	Patterned biomaterial/cell deposition	Serra and Piqué (2019), Delaporte et al. (2016)
Digital light projection (DLP)	Projected light pattern cures an entire hydrogel layer at once using DMD/LCD system	Photocurable hydrogels	Rapid printing, good resolution, uniform layer curing	Restricted to photoresponsive materials, possible photoinitiator/light toxicity	Tissue scaffolds with fine structural control	Jang et al. (2018), Ge et al. (2022)
3D plotting	Viscous hydrogel extruded through syringe/nozzle into supportive medium	Viscous hydrogels	Simple setup, controllable strand diameter, suitable for soft hydrogels	Lower resolution than light-based methods, requires matched support bath density	Soft scaffold fabrication	Jang et al. (2018), Puertas-Bartolomé et al. (2020)
Direct ink writing (DIW)	Continuous extrusion of shear-thinning ink through nozzle under pneumatic or mechanical pressure	Hydrogels, colloidal suspensions, bioinks	Broad material compatibility, easy multi-material printing, suitable for cell-laden inks	Resolution depends on rheology/nozzle size, shape collapse possible without fast recovery	Bioprinting of cartilage, bone, soft tissues	Advincula et al. (2021), Lewis, 2006; Ahn et al. (2011)
Low-temperature deposition manufacturing (LDM)	Extrusion onto subzero platform followed by freeze-drying	Temperature-sensitive hydrogels, polymer solutions	Useful for porous scaffold fabrication, avoids harsh thermal conditions	Freeze-drying step required, lower printing speed, limited fidelity for some soft inks	Porous tissue-engineering scaffolds	Liu et al. (2019)
Inkjet-based 3D printing with powder (I3DP-P)	Liquid binder selectively deposited onto powder bed layer by layer	Powder materials + binder	No need for additional support, relatively fast, versatile powder choice	Limited to low-viscosity binders, weaker mechanical strength of printed parts	Porous scaffold prototyping	Jang et al. (2018), Singh et al. (2010)
Inkjet-based 3D printing with liquid (I3DP-L)	Droplets of liquid ink or crosslinker are precisely deposited onto liquid/prepolymer system	Low-viscosity hydrogels, photosensitive polymers	High accuracy, non-contact deposition	Requires low viscosity, limited printable ink range, higher cost	Patterned hydrogel fabrication, fine bioprinting	Jang et al. (2018), Singh et al. (2010)

Although SLA, DLP, and extrusion-based methods are all widely used for hydrogel fabrication, their advantages are associated with clear trade-offs that should be considered when selecting a printing strategy for tissue engineering. In general, SLA and DLP provide higher printing resolution and better surface quality than extrusion-based methods, making them particularly suitable for fabricating constructs with fine architectural details. However, both techniques rely on photocurable materials and light exposure, which restrict the range of printable bioinks and may compromise cell viability because of photoinitiator toxicity, ultraviolet irradiation, or excessive crosslinking conditions. Among these light-based methods, DLP is generally faster than SLA because it cures an entire layer simultaneously rather than scanning point by point, although both remain less versatile than extrusion-based techniques in terms of printable material types. By contrast, extrusion-based methods such as 3D plotting and DIW are more compatible with viscous, shear-thinning, and cell-laden hydrogels, and therefore are often preferred for bioprinting applications. Their main limitations are lower resolution, filament spreading, and reduced structural fidelity, especially when the hydrogel lacks rapid recovery or sufficient mechanical stability after extrusion. Therefore, no single printing method is universally optimal: light-based printing is generally favored when structural precision is the priority, whereas extrusion-based printing is more advantageous when cytocompatibility, material diversity, and direct biofabrication of living constructs are the primary concerns (Huang J. et al., 2020; Jang et al., 2018; Ge et al., 2022; Lewis, 2006; Suntornnond et al., 2016; Ge et al., 2021).

**FIGURE 8:**
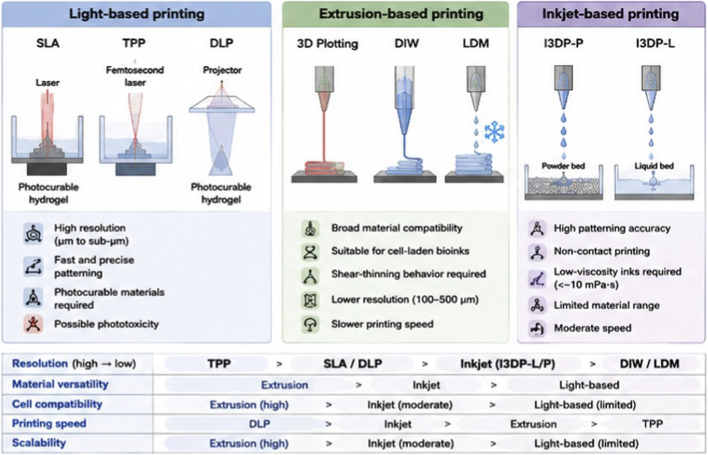
Comparative overview of major 3D printing techniques used for hydrogels in tissue engineering. Light-based printing methods, including SLA, TPP, and DLP, generally provide high structural resolution and precise patterning, but require photocurable materials and may involve phototoxicity-related limitations. Extrusion-based methods, such as 3D plotting, DIW, and LDM, offer broad material compatibility and are more suitable for viscous or cell-laden bioinks, although their resolution is typically lower and strongly dependent on rheological properties. Inkjet-based methods, including I3DP-P and I3DP-L, enable non-contact deposition and relatively high patterning accuracy, but are limited by low-viscosity material requirements and narrower material compatibility. The lower panel summarizes the practical trade-offs among resolution, material versatility, cell compatibility, printing speed, and scalability across these printing platforms.

As shown in Figure 8, the major hydrogel printing techniques used in tissue engineering can be broadly categorized into light-based, extrusion-based, and inkjet-based systems, each with distinct advantages, limitations, and application scenarios. Light-based printing methods, including SLA, TPP, and DLP, generally provide superior structural resolution and patterning precision because polymerization is spatially controlled by focused or projected light (Baker et al., 2012). Among these, TPP offers the highest spatial resolution, reaching the micro-to nanoscale, and is therefore particularly valuable for fabricating highly complex microarchitectures; however, its throughput and scalability remain limited (Huang J. et al., 2020; Jang et al., 2018; O'Halloran et al., 2023). SLA also enables precise layer-by-layer fabrication with smooth surface quality, but its point-by-point laser scanning process is relatively slow and depends strongly on photocurable materials (Huang J. et al., 2020; Chua and Leong, 2014; Jang et al., 2018). DLP, in contrast, cures an entire layer simultaneously and is therefore generally faster than SLA while still maintaining good structural fidelity (Jang et al., 2018; Ge et al., 2022). Nevertheless, both SLA and DLP are restricted by the need for photoresponsive hydrogel systems and may raise cytocompatibility concerns related to photoinitiator toxicity, ultraviolet irradiation, or excessive crosslinking conditions (Huang J. et al., 2020; Jang et al., 2018; Ge et al., 2022).

Extrusion-based methods, such as 3D plotting, DIW, and LDM, offer broader material compatibility and are more suitable for viscous or cell-laden bioinks (Jang et al., 2018; Lewis, 2006; Suntornnond et al., 2016; Liu et al., 2019). These methods are especially attractive in tissue engineering because they can process shear-thinning hydrogel formulations and enable direct deposition of biologically relevant materials, including cells, colloids, and composite bioinks (Lewis, 2006; Suntornnond et al., 2016). In DIW, for example, printability depends strongly on rheological properties such as viscosity, shear-thinning behavior, yield stress, and post-extrusion shape recovery, all of which influence filament fidelity and structural stability (Lewis, 2006; Suntornnond et al., 2016). Similarly, 3D plotting is useful for soft hydrogel systems, although it often requires a density-matched support medium and provides lower resolution than light-based approaches (Jang et al., 2018; Puertas-Bartolomé et al., 2020). LDM extends extrusion-based printing by combining low-temperature deposition with freeze-drying, which is advantageous for fabricating porous scaffolds and avoiding harsh thermal conditions, but it is less straightforward for direct cell-laden applications because of subzero processing requirements (Liu et al., 2019; Singh et al., 2010). Overall, extrusion-based platforms are generally favored when material versatility, cytocompatibility, and direct biofabrication are prioritized, even though their spatial resolution is usually lower than that of light-based systems (Jang et al., 2018; Lewis, 2006; Suntornnond et al., 2016; Liu et al., 2019). Inkjet-based methods, including I3DP-P and I3DP-L, provide non-contact deposition and relatively high patterning accuracy, making them useful for fine hydrogel placement and rapid prototyping (Jang et al., 2018; Ge et al., 2021; Vieira et al., 2017). In I3DP-P, the surrounding unbound powder can serve as a temporary support material, which is advantageous for shape maintenance during fabrication (Jang et al., 2018; Ge et al., 2021). In contrast, I3DP-L relies on the deposition of droplets of liquid hydrogel precursor or crosslinking solution, enabling localized gelation with good positional accuracy (Jang et al., 2018). However, both inkjet-based approaches are limited by the requirement for low-viscosity printable formulations, which narrows the range of compatible hydrogel systems and may restrict their use for highly viscous or mechanically robust bioinks (Jang et al., 2018; Ge et al., 2021). Therefore, while inkjet printing offers attractive advantages in accuracy and non-contact operation, its material range and mechanical outcomes are typically more constrained than those of extrusion-based systems (Jang et al., 2018; Ge et al., 2021; Vieira et al., 2017).

Taken together, Figure 8 highlights that no single printing technique is universally optimal for all hydrogel-based tissue-engineering applications. Instead, the choice of method should be guided by the required balance among structural resolution, material versatility, cytocompatibility, printing speed, and scalability (Huang J. et al., 2020; Jang et al., 2018; Ge et al., 2022; Lewis, 2006; Suntornnond et al., 2016; Ge et al., 2021). Light-based methods are generally preferred for applications requiring fine architectural control, extrusion-based methods are more suitable for cell-laden and rheologically complex bioinks, and inkjet-based methods are advantageous when precise, non-contact deposition of low-viscosity materials is needed. This comparative framework is important for matching the printing platform to the biological and mechanical demands of the target tissue.

## 3 Natural hydrogels

Naturally derived hydrogels can be divided into three categories: polysaccharide-based, protein-based, and those derived from decellularized tissue. These hydrogels are naturally bioactive and biocompatible, as they contain proteins and extracellular matrix (ECM) components. As a result, they have demonstrated considerable potential in biomedical applications, as they can promote cellular functions. However, these hydrogels have limitations, including challenges in manipulation and high variation between batches (Vieira et al., 2017).

**FIGURE 9:**
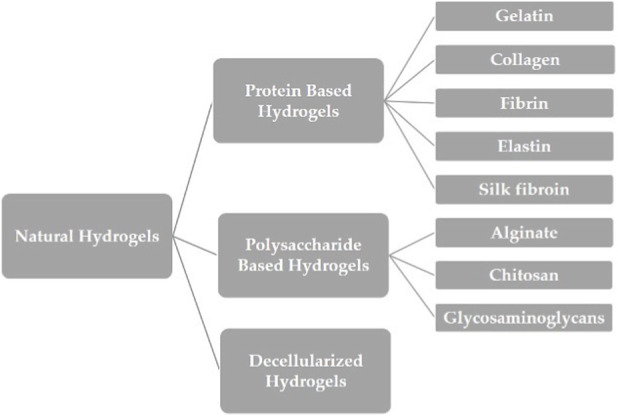
Schematic classification of natural hydrogels used in tissue engineering. Natural hydrogels are grouped according to their biological origin and major material categories, including protein-based, polysaccharide-based, and other naturally derived systems. This classification highlights the diversity of natural hydrogel materials and their distinct biological and structural characteristics relevant to 3D bioprinting applications (Catoira et al., 2019).

Each hydrogel type has distinct properties that make it suitable for specific applications. The various natural materials used to create hydrogels have unique features that are worth exploring in more detail. Figure 9 provides an overview of the different materials, including gelatin, elastin, collagen, silk fibroin, and fibrin. collagen, elastin, and fibrin are proteins commonly found in the ECM structure, providing the necessary strength and elasticity for proper tissue function (Catoira et al., 2019). By classifying these materials according to their biological origin and major structural characteristics, Figure 9 emphasizes that natural hydrogels are not a single uniform group, but rather a broad family of biomaterials with distinct bioactivities, processing behaviors, and mechanical limitations. This distinction is important because the suitability of each material for 3D printing and tissue-specific applications depends strongly on these underlying differences. These proteins are typically sourced from animal-derived tissues, such as porcine tissue or murine tails, while fibroin is obtained from insects. Natural hydrogels are ideal for cell culture systems and tissue engineering, with significant potential. Nevertheless, these hydrogels still present several limitations, including challenges with manipulation and high batch-to-batch variation (Vieira et al., 2017).

### 3.1 Alginate hydrogels

Alginate is a natural polysaccharide that originates from marine sources and is commonly used as a thickener, stabilizer, and texturizer in the food industry (Bam et al., 2019). Recently, alginate has garnered significant attention in the biomedical field, particularly in the development of functional foods. Alginate-based biomaterials have been utilized in tissue engineering to repair soft and hard tissues such as skin, bone, heart, cartilage, and vascular tissue (Bidarra et al., 2011). To improve the mechanical, biological, and structural properties of polymer-based biomaterials, chemical modification is often employed. Alginate is highly hydrophilic and exhibits low protein adsorption, making it a non-fouling biomaterial (Morra and Cassineli, 1999). Despite its lack of cell-interactive domains, it can be a beneficial biomaterial in certain cases. Non-modified alginate can be altered chemically to promote specific biological responses in a highly regulated manner (Lee and Mooney, 2012).

### 3.2 Chitosan hydrogels

Chitosan is a natural polysaccharide that is commonly used to create biocompatible hydrogels (Suntornnond et al., 2016). It is a natural polymer that is derived from chitin. Chitin is found in the exoskeletons of insects, crustaceans, and mollusks, as well as in the cell walls of fungi (Younes and Rinaudo, 2015). Chitosan’s structure is composed of d-glucosamine and N-acetylglucosamine units that are linked together through β(1–4) glycosidic bonds. Chitosan’s polysaccharide structure contains glucosamine and N-acetylglucosamine, with glucosamine being a product of glucose in the body that can produce glucosaminoglycans (GAGs), a component of the extracellular matrix and cartilage tissue (Baldrick, 2010).

Recent research has shown that chitosan-based tissue scaffolds consist of 14.75% free chitosan and 85.25% chitosan in a combined form (Ribeiro et al., 2017), and their pH can be adjusted or can be dissolved in a nonsolvent to create a gel (Xu et al., 2017; Peppas et al., 2006). Combining chitosan with other polymers, such as hyaluronic acid, can result in thermosensitive hydrogels through electrostatic interactions, but physically crosslinked hydrogels can have inadequate mechanical properties and uncontrolled dissolution. To overcome these limitations, chemical modifications of chitosan have been explored to improve its properties and expand its potential applications (Zhang et al., 2019).

### 3.3 Fibrin hydrogels

When healing a wound caused by trauma or other tissue damage, cells encounter a fibrin network scaffold, made of thrombin and fibrinogen, that was one of the first biomaterials used to promote wound healing and prevent bleeding. The unique polymerization mechanism of fibrin can precisely control the gelation time and network structure, thus enabling the formation of various soft matrices under physiological conditions (Weisel and Litvinov, 2013). This substrate has been extensively studied in rheology due to its nonlinear elasticity, characterized by soft compliance at small strains and rigidity against larger deformations, allowing it to deform significantly without fracture. This elasticity is critical for their role in wound healing, cell migration, and tampons, and has potential applications in bioengineering and medicine (Janmey et al., 2009).

### 3.4 Cellulose hydrogels

Cellulose has numerous advantages such as biocompatibility, biodegradability, renewability, good mechanical strength, eco-friendliness, and safety, making it one of the most attractive materials for various applications (Chen et al., 2015). Due to its high intermolecular and intramolecular hydrogen bonds and van der Waals forces, it is tasteless, odorless, and insoluble in water or most organic solvents. Moreover, their applications in hydrogel fabrication are becoming increasingly widespread due to their high mechanical strength, biodegradability, biocompatibility, and environmental friendliness. In the preparation of cellulose hydrogels, in order to use cellulose as a base to make hydrogels, various factors must be considered, such as the dissolution and preparation of cellulose and the techniques used to produce hydrogels. The technique can employ physical or chemical crosslinking, polymerization methods, or a mixture of techniques and can be selected according to the specific application (Zainal et al., 2021).

## 4 Synthetic hydrogels

In contrast to natural hydrogels, synthetic hydrogels offer greater control over chemical composition, molecular architecture, and mechanical properties, making them attractive materials for 3D bioprinting and tissue engineering applications. Although natural hydrogels provide favorable bioactivity and biocompatibility, they often lack the mechanical robustness required for many applications. Synthetic polymers, by comparison, can be more precisely designed to achieve desired structural and physicochemical properties (Radulescu et al., 2022).

The use of synthetic polymers in the fabrication of 3D printed polymer hydrogels has several advantages over natural hydrogels. When producing synthetic polymers, a wide variety of monomers and crosslinkers can be selected for specific qualities such as degree of functionalization, molecular weight, and surface shape, and by varying these properties, specific synthetic polymers can be tailored for specific purposes, such as polyvinyl alcohol (PVA), PEG and polyacrylamide derivatives, etc (Bashir et al., 2020).

Although synthetic hydrogels have their advantages, such as controllable chemical and physical properties, they also have limitations when it comes to biocompatibility and biodegradability. Synthetic hydrogels produced by chain-growth addition polymerization may produce highly crosslinked structures with elevated viscosity and residual unreacted monomers, which can hinder full consumption of monomers and leave leftover, frequently contaminated monomers in the structure (Sánchez-Cid et al., 2022). The ability to sterilize hydrogels can be impacted by their synthetic nature and the presence of residual water molecules, which can complicate the process. Moreover, the breakdown rate of synthetic polymer hydrogels used in regenerative medicine may not be in line with their original forms, raising concerns about their biodegradability (Advincula et al., 2021).

Synthetic polymer hydrogels can be classified into three categories based on their preparation methods: homopolymer hydrogels, copolymer hydrogels, and multipolymer hydrogels. Another type, called interpenetrating polymer hydrogels, is formed by creating a first network and then swelling it in another monomer to form a second intermeshing network (Ahmad et al., 2022). Examples of synthetic hydrogels include PVA, PEG, polycaprolactone (PCL), polyglycolic acid (PGLA), polylactic acid (PLA), polyacrylates, polyvinylpyrrolidone (PVP), polyimide (PI), PEO, and their derivatives (Thang et al., 2023).

### 4.1 pHEMA, or poly (hydroxyethyl methacrylate) hydrogels

**FIGURE 10:**
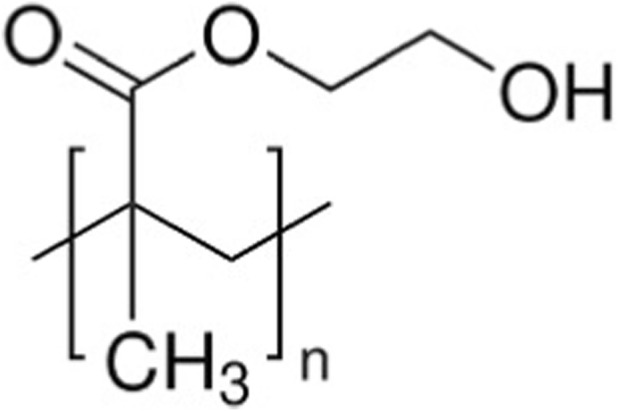
Repeating unit structure of poly (2-hydroxyethyl methacrylate) (pHEMA) (Topuz et al., 2018).

Poly (hydroxyethyl methacrylate) (pHEMA) is a synthetic polymer capable of forming hydrogels in aqueous environments, and its chemical structure is shown in Figure 10. The structure of pHEMA helps explain its hydrogel-forming ability and hydrophilic behavior, which underlie its long-standing use in biomedical applications. In particular, the hydrophilic functional groups facilitate water uptake and soft-tissue compatibility, whereas the methacrylate backbone enables polymerization, network formation, and structural stability. These structure–property relationships make pHEMA suitable for a variety of biomedical products, most notably contact lenses. pHEMA-based contact lenses are commonly prepared by solution polymerization of hydroxyethyl methacrylate (HEMA) using initiators such as sodium metabisulfite and ammonium persulfate, and may also include crosslinking agents such as triethylene glycol dimethacrylate. Beyond contact lenses, pHEMA has also shown potential in other biomedical applications, including artificial skin and wound-healing materials. (Topuz et al., 2018; Zare et al., 2021).

### 4.2 PEG or poly (ethylene glycol) hydrogels

**FIGURE 11:**
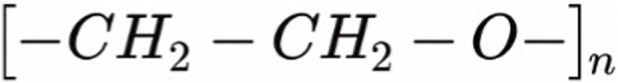
Representative chain structure of polyethylene glycol (PEG) (Topuz et al., 2018).

Polyethylene glycol hydrogel, also known as PEG hydrogel, is a commonly used synthetic polymer hydrogel in the medical and biomedical fields due to its excellent biocompatibility. The chemical structure of PEG (Figure 11) can be represented by the formula HO(CH_2_CH_2_O)nH (Topuz et al., 2018). As shown in Figure 11, the repeating ethylene glycol units confer strong hydrophilicity and flexibility to PEG, which explains its excellent water solubility and resistance to nonspecific protein adsorption. These structural features are closely related to the widespread use of PEG in biomedical hydrogels, particularly in applications requiring biocompatibility, tunable network formation, and controlled delivery of biomolecules. Therefore, Figure 11 helps connect the simple chemical structure of PEG with its important functional role as a synthetic hydrogel platform. PEG is a kind of hydrophilic polymer, which is very soluble in water and not easy to degrade. Due to its hydrophilic nature, PEG is highly resistant to protein adsorption and cell adhesion, and it is a commonly used synthetic polymer in the biomedical field for the manufacture of polymeric medical devices and regenerative medicine products (Lin and Anseth, 2009). It has a wide range of applications, including wound healing and cell culture research, and it is also widely used as an ingredient in mineral oils, alcohol products, hydraulic fluids, and various other commercial products (Topuz et al., 2018).

### 4.3 PVA (poly (vinyl alcohol)) hydrogels

**FIGURE 12:**
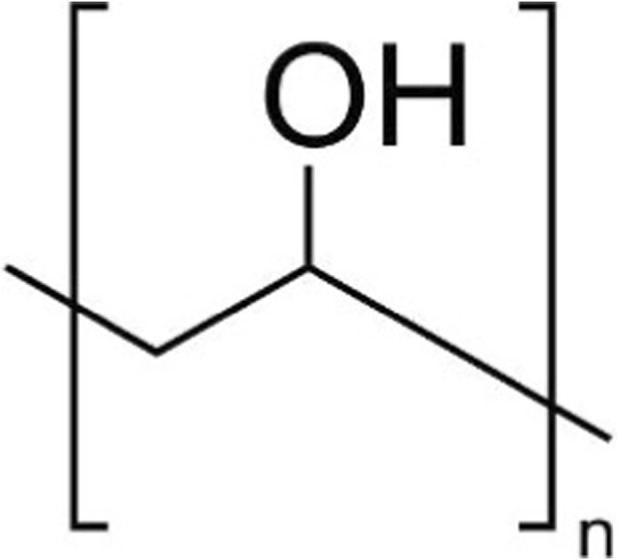
Repeating unit structure of poly (vinyl alcohol) (PVA) (Jang et al., 2018).

PVA, a synthetic polymer that is soluble in water, has a molecular formula of [CH_2_CH(OH)]n or (C_2_H_4_O)_x_, as depicted in Figure 12 (Jang et al., 2018; Karmaker et al., 2021). Figure 12 illustrates the hydroxyl-rich structure of PVA, which is directly related to its water solubility, hydrogen-bonding capacity, and favorable mechanical properties in hydrogel form. These structural features help explain why PVA hydrogels often show good shape recovery, bulk stability, and utility in biomedical applications such as wound healing and cartilage-related materials. Thus, the figure reinforces the connection between PVA’s polymer chemistry and its practical value in hydrogel engineering. Its colorless and odorless properties make it suitable for use in various industries, such as textiles, papermaking, and coatings. Additionally, PVA has a range of applications, including in regenerative medicine, due to its biocompatibility and hydrophilic nature. It is commonly supplied in the form of solid beads or a solute that dissolves in water. In the biomedical field, cross-linked PVA hydrogels have numerous applications such as wound healing, cartilage regeneration, contact lenses, and synthetic organs (Baker et al., 2012). Furthermore, non-modified PVA hydrogels do not adhere to cells or proteins, making it an exceptional material for tissue engineering (Wang et al., 2021). PVA hydrogels are known to exhibit enhanced mechanical stiffness and the ability to regain their original shape in water, which contributes to their superior bulk properties. These hydrogels can be produced using various methods, such as freezing and thawing, which result in higher mechanical rigidity compared to those produced through other methods such as UV light cross-linking (Topuz et al., 2018).

Given the wide diversity of hydrogel materials used in 3D printing, a comparative overview of the main hydrogel types is provided in Table 2. Natural hydrogels, such as alginate, chitosan, fibrin, and cellulose-based systems, are generally favored for their excellent biocompatibility and biomimetic characteristics, but they often suffer from limited mechanical strength and batch variability. In contrast, synthetic hydrogels, including pHEMA, PEG, and PVA, offer better control over chemical composition and mechanical properties, although they usually lack intrinsic bioactivity and may require further functional modification. In addition, tough and composite hydrogels have emerged as important material systems because they combine improved mechanical robustness with tunable biological and printing performance. Therefore, the selection of hydrogel type should be based on a balance between printability, mechanical stability, biological functionality, and the requirements of the target tissue application.

**TABLE 2:** Comparison of hydrogel types used in 3D printing for tissue engineering.

Hydrogel type	Representative materials	Key advantages	Main limitations	Typical 3D printing compatibility	Representative tissue engineering uses	Refs
Natural hydrogels	Alginate, chitosan, fibrin, cellulose	Excellent biocompatibility, bioactivity, ECM-like environment, biodegradable	Weak mechanical strength, batch-to-batch variation, difficult processing control	Mainly extrusion-based printing; some formulations adaptable to inkjet/light-assisted methods	Skin, bone, cartilage, vascular tissue	Vieira et al. (2017), Catoira et al. (2019)
Alginate-based hydrogels	Alginate and modified alginate composites	Mild gelation, easy processing, good printability, widely used in bioinks	Limited cell adhesion unless modified, relatively weak bioactivity	DIW, extrusion bioprinting, inkjet-related systems	Soft tissue, vascular, cartilage, wound healing	Bam et al. (2019), Bidarra et al. (2011), Morra and Cassineli (1999), Lee and Mooney (2012), Wei et al. (2023), Ilhan et al. (2020), Tytgat et al. (2019)
Chitosan-based hydrogels	Chitosan and chemically modified chitosan	Biocompatible, biodegradable, antimicrobial, bioactive polysaccharide structure	Poor solubility at physiological pH, often inadequate mechanics without modification	Mainly extrusion/DIW; blends often needed	Tissue scaffolds, drug delivery, wound healing	Younes and Rinaudo (2015), Baldrick (2010), Ribeiro et al. (2017), Xu et al. (2017), Peppas et al. (2006), Zhang et al. (2019), Rajabi et al. (2021), Taghizadeh et al. (2022)
Fibrin hydrogels	Fibrinogen/thrombin-derived gels	Physiological gelation, excellent cell compatibility, supports wound healing and migration	Weak long-term stability, rapid degradation	Bioprinting and soft matrix fabrication	Wound healing, cell migration scaffolds	Weisel and Litvinov (2013), Janmey et al. (2009)
Cellulose-based hydrogels	Cellulose derivatives, nanocellulose, TOCNF	Good mechanical strength, biodegradability, biocompatibility, rheology enhancement	Difficult dissolution/processing, often requires derivatization	Particularly suitable for extrusion/DIW bioinks	Cartilage, bone, reinforcement of bioinks	Chen et al. (2015), Zainal et al. (2021), Wang et al. (2021), Wang et al. (2018), Dai et al. (2019), Markstedt et al. (2015), Ávila et al. (2016), Im et al. (2022)
Synthetic hydrogels	pHEMA, PEG, PVA, PAAm derivatives	Tunable chemistry, controllable mechanical/physical properties, reproducible manufacture	Lower intrinsic bioactivity, possible concerns over residual monomers/degradation products	Broad compatibility depending on formulation; strong potential for light-based and extrusion printing	General scaffold engineering, controlled systems	Radulescu et al. (2022), Bashir et al. (2020), Sánchez-Cid et al. (2022), Ahmad et al. (2022), Thang et al. (2023)
pHEMA hydrogels	Poly (hydroxyethyl methacrylate)	Good hydrogel-forming ability, established biomedical use	Limited intrinsic bioactivity, often requires modification for tissue engineering	Photo/polymerization-based fabrication	Contact lenses, artificial skin-related applications	Zare et al. (2021)
PEG hydrogels	PEG and PEG derivatives	Excellent biocompatibility, hydrophilic, highly tunable, resistant to protein adsorption	Poor inherent cell adhesion, often requires biofunctionalization	Particularly suitable for SLA/DLP and other photocrosslinkable systems	Controlled release, regenerative medicine, cell culture	Lin and Anseth (2009)
PVA hydrogels	Poly (vinyl alcohol)	Good hydrophilicity, biocompatibility, mechanical robustness, shape recovery	Low intrinsic cell adhesion, often requires crosslinking optimization	Extrusion and molded hydrogel systems; some printable composite formulations	Cartilage, wound healing, artificial organs	Baker et al. (2012), Karmaker et al. (2021), Wang et al. (2021)
Tough/composite hydrogels	Nanocomposite, supramolecular, double-network, gradient, hydrogel–polymer hybrids	Improved toughness, multifunctionality, enhanced printability and stability	More complex formulation and fabrication, sometimes multi-step curing	Especially relevant to DIW/extrusion and advanced hybrid printing	Bone, cartilage, load-bearing and functional scaffolds	Ge et al. (2021), Zorlutuna et al. (2012), Kolesky et al. (2016), Guo et al. (2020), Yu et al. (2019), Zhu et al. (2017), Zhai et al. (2017), Wahid et al. (2020), Hong et al. (2015), Song and Wang (2020), Wei et al. (2015), Gao et al. (2019), Gungor-Ozkerim et al. (2018)

**FIGURE 13:**
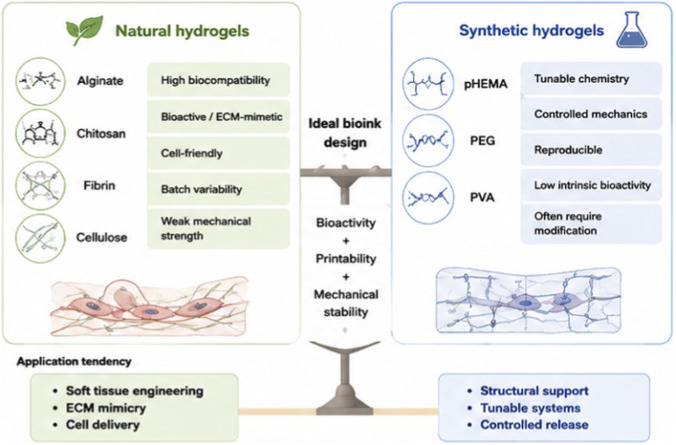
Comparison of natural and synthetic hydrogels used in 3D bioprinting. Natural hydrogels, including alginate, chitosan, fibrin, and cellulose-based systems, are generally characterized by high biocompatibility, bioactivity, ECM-mimetic properties, and cell-friendly behavior, but they often show batch-to-batch variability and limited mechanical strength. In contrast, synthetic hydrogels, such as pHEMA, PEG, and PVA, offer tunable chemistry, controlled mechanical properties, and improved reproducibility, although they typically exhibit lower intrinsic bioactivity and often require further modification. The central balance scale illustrates that ideal bioink design requires an appropriate balance among bioactivity, printability, and mechanical stability. The lower panels summarize the general application tendencies of natural hydrogels toward soft tissue engineering, ECM mimicry, and cell delivery, and of synthetic hydrogels toward structural support, tunable systems, and controlled release.

As shown in Figure 13, natural and synthetic hydrogels possess distinct but complementary characteristics in 3D bioprinting and tissue engineering. Natural hydrogels, such as alginate, chitosan, fibrin, and cellulose-based systems, are generally favored for their high biocompatibility, bioactivity, and extracellular matrix (ECM)-mimetic properties, which make them particularly suitable for soft tissue engineering, cell delivery, and the creation of cell-friendly microenvironments (Jang et al., 2018; Bam et al., 2019; Bidarra et al., 2011; Baldrick, 2010; Chen et al., 2015; Bashir et al., 2020). However, these materials often exhibit limited mechanical strength and batch-to-batch variability, which may restrict their structural stability and reproducibility in printed constructs (Bam et al., 2019; Bidarra et al., 2011). In contrast, synthetic hydrogels, including pHEMA, PEG, and PVA, provide greater control over chemical composition, mechanical properties, and reproducibility, making them attractive for applications requiring structural support, tunable material performance, and controlled release (Baker et al., 2012; Sánchez-Cid et al., 2022; Ahmad et al., 2022; Thang et al., 2023; Zare et al., 2021; Lin and Anseth, 2009; Wang et al., 2021; Balakrishnan and Banerjee, 2011; Wang et al., 2018). Nevertheless, their lower intrinsic bioactivity means that additional modification is often needed to improve cell interactions and biological functionality (Baker et al., 2012; Sánchez-Cid et al., 2022; Ahmad et al., 2022; Wang et al., 2018). Therefore, this figure highlights that ideal bioink design should not rely solely on either natural or synthetic systems, but rather on achieving an appropriate balance among bioactivity, printability, and mechanical stability according to the requirements of the target tissue application.

## 5 The application of tough hydrogel for 3D bio-printing

**FIGURE 14:**
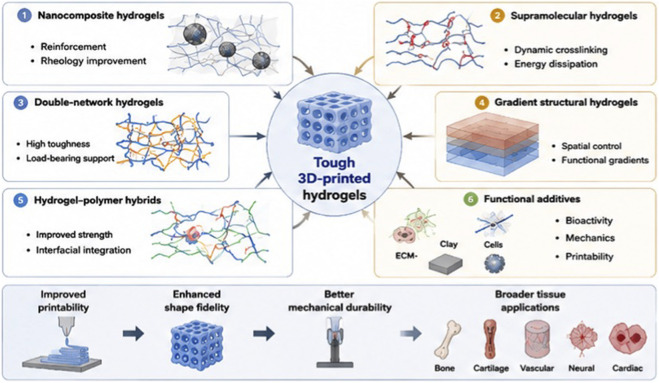
Toughening strategies for 3D-printed hydrogels. Multiple design approaches can be used to improve the mechanical performance and functional utility of hydrogel-based printed constructs, including nanocomposite hydrogels, supramolecular hydrogels, double-network hydrogels, gradient structural hydrogels, hydrogel–polymer hybrids, and the incorporation of functional additives. These strategies enhance key properties such as reinforcement, rheological behavior, dynamic crosslinking, load-bearing capacity, spatial control, interfacial integration, bioactivity, and printability. As summarized in the lower panel, these improvements contribute to better printability, enhanced shape fidelity, greater mechanical durability, and broader tissue-engineering applications, including bone, cartilage, vascular, neural, and cardiac tissues.

As shown in Figure 14, the mechanical limitations of conventional hydrogels can be addressed through multiple toughening strategies, each contributing to the performance of 3D-printed constructs through different mechanisms. Nanocomposite hydrogels improve reinforcement and rheological behavior through the incorporation of nanoscale fillers, which can enhance both printability and structural stability (Zorlutuna et al., 2012; Kolesky et al., 2016). Supramolecular hydrogels rely on reversible noncovalent interactions, such as hydrogen bonding or host–guest interactions, to promote dynamic crosslinking and energy dissipation under mechanical stress (Guo et al., 2020). Double-network hydrogels, composed of interpenetrating polymer networks, are particularly effective in improving toughness and load-bearing capacity, making them attractive for mechanically demanding tissue-engineering applications (Yu et al., 2019). Gradient structural hydrogels further expand functional design by enabling spatial control over composition and bioactivity, which is especially valuable in osteochondral and other interface tissues requiring region-specific properties (Zhu et al., 2017). In addition, hydrogel–polymer hybrids can improve overall strength and interfacial integration by combining the favorable hydration and biological features of hydrogels with the structural advantages of polymeric components (Catoira et al., 2019; Zhai et al., 2017). Functional additives, including extracellular matrix components, living cells, nanoclays, and bioactive particles, can also be incorporated to simultaneously improve bioactivity, mechanics, and printability (Wahid et al., 2020; Wang et al., 2023; Zhou et al., 2024). Taken together, these strategies contribute to improved printability, enhanced shape fidelity, better mechanical durability, and broader applicability of hydrogel-based constructs in bone, cartilage, vascular, neural, and cardiac tissue engineering.

### 5.1 Biopolymer-based tough hydrogels

**FIGURE 15:**
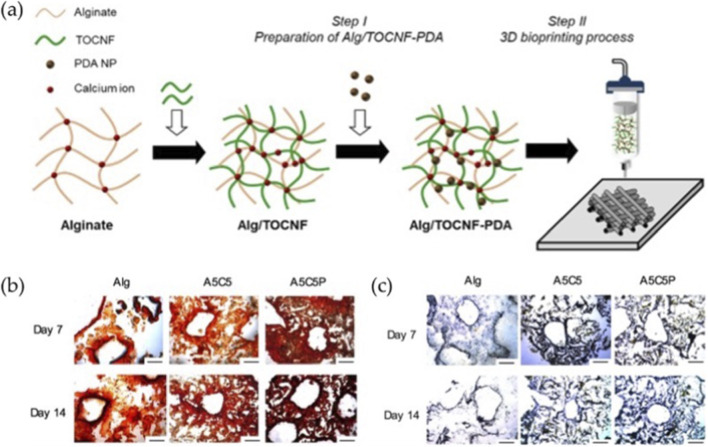
Representative osteogenic performance of a composite bioink for 3D bioprinting and bone tissue engineering. **(a)** Schematic illustration of the preparation of a composite bioink containing alginate, cellulose nanofibrils, and polydopamine nanoparticles for 3D bioprinting. **(b)** Alizarin red staining and **(c)** Von Kossa staining images showing mineralization in printed scaffolds. These results demonstrate the potential of composite bioink design to enhance osteogenic activity while maintaining printability and structural support. Scale bars: 50 μm (Im et al., 2022).

Biopolymers, which are mainly derived from living organisms including microbes, plants, and animals, that increasingly popular in drug delivery, bioengineering, and food industry due to their excellent bioavailability, biocompatibility, and biodegradability (Balakrishnan and Banerjee, 2011). Natural polysaccharides such as cellulose (Wang et al., 2018; Dai et al., 2019), sodium alginate (Wei et al., 2023; Ilhan et al., 2020), κ-carrageenan (Tytgat et al., 2019; Yu et al., 2022), and chitosan (Rajabi et al., 2021; Taghizadeh et al., 2022) are commonly employed to engineer biopolymer hydrogels on 3D printing techniques over the past decade. Cellulose, the most abundant polysaccharide found in forestry products, is commonly form tough hydrogels in 3D printing process (Markstedt et al., 2015). This is due to the numerous hydroxyl groups present in cellulose that can create intra- and inter-chain hydrogen bonds, resulting in strong gels and enhance the mechanical properties. Various cellulose derivatives, such as microcrystalline cellulose, cellulose ethers/esters, and nanocellulose, have been utilized for 3D printing due to their high melt viscosity and limited solubility. For example, Gatenholm et al. combined alginate with quick gelation ability and nano-fibrillated cellulose with shear-thinning behavior to produce bio-ink for inkjet-based 3D printing for cartilage tissue engineering (Markstedt et al., 2015; Ávila et al., 2016). Additionally, Im et al. formulated various hydrogel-based bioinks using natural and biocompatible biomaterials (i.e., alginate, tempo-oxidized cellulose nanofibrils (TOCNF), and polydopamine nanoparticles (PDANPs) for 3D bioprinting and bone tissue engineering (Im et al., 2022). As shown in Figure 15, the combined use of alginate, cellulose nanofibrils, and polydopamine nanoparticles represents a representative strategy for overcoming the limitations of single-component hydrogel systems. The figure not only demonstrates the formulation process of the composite bioink, but also links material design to biological outcome by showing enhanced mineralization in the printed scaffolds. This example highlights how bioink composition can be rationally tailored to improve printability, mechanical support, and osteogenic performance simultaneously in bone tissue engineering.

In certain cases, hydrogel composed of a sole biopolymer may not have sufficient mechanical strength to meet the practical 3D printing application demands. To tackle this issue, a composite system can be established by adding a small amount of synthetic polymer in biopolymer, leading to 3D printed hydrogels with improved strength. Combining biopolymers with synthetic polymers, such as PVA, PEG, PAAm, PNIPAm, etc., is a common approach to enhance the mechanical properties of biopolymer hydrogels. This is achieved by forming double-component inks that can be used for 3D printing (Hong et al., 2015).

### 5.2 Synthetic single-network tough hydrogels

#### 5.2.1 Tough nanocomposite hydrogels

Nanocomposite hydrogels with a specialized polymer network structure and dispersed nanoparticles that crosslinks within the gel matrix and provide multifunctional properties. The nanoparticles can be categorized based on their composition as metallic, organic, or inorganic, and their shape as spherical, flaky, or fibrous. These hybrid materials due to their unique properties that have promising applications in various fields. The particles, which are typically stiff and may undergo partial orientation under shear force during the 3D printing extrusion step, are effective at toughening the hydrogels. Incorporating even a small amount of nano-clays with hydrogels can yield significant improvements in both the mechanical properties of hydrogels and the rheological behavior of 3D printing inks. As a result, this technique holds great promise for expanding the possibilities of 3D printing technology in the field of engineering (Wahid et al., 2020).

#### 5.2.2 Tough supramolecular hydrogels

Supramolecular biopolymers (SBPs) refer to polymeric units that are formed from macromolecules and have the ability to self-assemble through noncovalent interactions such as metal coordination bonds, ionic bonds, hydrogen bonds, and hydrophobic interactions (Song and Wang, 2020). These independent units can interact with each other to form intricate and large structural networks. Supramolecular hydrogels mean that polymer chains of SBPs are linked physically by non-covalent bonds. These hydrogels are toughened by utilizing various noncovalent bonds (Song and Wang, 2020).

It should be emphasized that the use of both synthetic polymers and biopolymers played a crucial role in the emergence of tissue engineering. Synthetic polymers enabled the creation of 3D models that mimicked the shapes of natural tissues, highlighting the limitations of traditional two-dimensional cell cultures in replicating the properties of most tissues. Biopolymers, on the other hand, provided materials with inherent biocompatibility and biodegradability, facilitating the development of functional tissue constructs (Zorlutuna et al., 2012).

#### 5.2.3 Conducting and responsive tough hydrogels

Strong hydrogels not only with exceptional mechanical properties also possess distinct functionalities by employed specific polymer that can be customized through 3D printing to suit specific requirements. In this context, there are two types of 3D-printed functional hydrogels that are of particular interest - conducting tough hydrogels and stimuli-responsive tough hydrogels. Conducting polymers are known for their conjugated backbones, which give them inherent electrical conductivity. This property can be incorporated into hydrogels to form tough hydrogels with applications in areas such as tissue engineering, biosensors, and energy storage. On the other hand, stimuli-responsive tough hydrogels can undergo reversible molecular interaction changes in response to external stimuli like temperature, pH, or light. These hydrogels can be designed to be responsive to specific triggers, allowing for their use in drug delivery, bioelectronics, energy-related devices, flexible electronics, tissue engineering, and other biomedical applications (Guo et al., 2020).

### 5.3 Composite structure hydrogels

#### 5.3.1 Double-network hydrogels

Researchers have explored double network (DN) hydrogels with remarkable reinforcement mechanism and good printability to achieve superior mechanical properties for 3D printed structures. However, DN hydrogels given their relatively complex network structure, compared to the single-network hydrogels mentioned above, that usually require multiple polymerization steps for 3D printing. Carrageenan with temperature-induced sol-gel transition property that utilized to initiate the initial gelling of the extruded hydrogel ink. After the initial printing of the hydrogel, a photo initiator and unreacted AAm monomer were exposed to UV light to create a second PAAm network, resulting in a DN hydrogel structure. This new hydrogel structure exhibited superior mechanical properties compared to PAAm gel or single-network carrageenan gel. Despite this, the long-term stability of the printed DN gel was found to be inadequate when immerse in aqueous solution, potentially due to the osmotic pressure damaging the helix structure between -carrageenan chains (Wei et al., 2015).

#### 5.3.2 Gradient structural hydrogels

Gradient structural materials are commonly found in natural organisms, providing of distinctive mechanical and biological functionalities. Developing hydrogel materials with precisely engineered gradient structures is crucial for meeting specific application requirements. 3D printing with gradient structural hydrogel has gained recognition as a highly precise and customizable processing technology to achieve various objectives.

To enhance the effectiveness of tissue repair, a hydrogel scaffold with a gradient structure was developed, which utilizes the benefits of bioactive Mn^2+^ ions incorporated into the top cartilage layer, and bioactive glass (BG) integrated into the bottom subchondral bone layer. Utilizing 3D printing technology, this gradient hydrogel scaffold was crafted to achieve highly precise and customizable structures. The incorporation of Mn2+ ions and BG within the scaffold promoted the differentiation of bone marrow stem cells into osteogenic and chondrogenic lineages, respectively (Gao et al., 2019).

#### 5.3.3 Hydrogel-polymer hybrids

Hydrogel-polymer hybrid materials are usually synthesized by combining hydrogels with polymers, and they have a wide range of application potential in various fields (Yu et al., 2019). Although their material differences make it challenging to achieve strong interfacial adhesion between these materials, advanced fabrication techniques enable precise control over the shape and structure of hydrogel-polymer mixtures. One method to achieve this involves 3D printing using extrusion, which allows for the creation of tough hydrogel-elastomer hybrids with delicate structures. During the curing process, an interlink initiator is added to the printing ink, forming covalent bonds between the hydrogel and the elastomer network, resulting in a superiorly tough printed hybrid. Furthermore, the hydrogel’s ability to be applied in air is made possible by adding a hydrophobic elastomer coating, which enhances its water retention capacity (Ge et al., 2021).

#### 5.3.4 Macroscopic topological designed hydrogels

By thoughtfully designing macroscopic structures, the overall toughness of materials can be substantially improved. Utilizing of 3D printing technology, hydrogel structures can be precisely tailored to improve their mechanical properties.

Multiple nozzles of different diameters are used to produce gel fibers at different locations. The printed structure was crafted using thick filaments approximately 0.4 mm wide, providing a solid base. Additionally, slender filaments with a diameter of approximately 0.2 mm were incorporated as “flexible bonds” situated in the folded domains. This clever design allowed for the thinner fibers to break initial when exposed to a load. This action subsequently freed up the concealed length of the folded domains, thereby bolstering the overall extensibility and durability of the primary framework (Zhu et al., 2017).

### 5.4 Functional additives in printed tough gels

#### 5.4.1 Rheology modifiers

The ability to print with hydrogel ink hinges greatly on its rheological properties, which directly impact the number and quality of the printed structures. It is imperative for the printing ink to exhibit the appropriate viscosity to ensure continuous extrusion and maintain its shape without undesired spreading. To expand the applicability of hydrogel materials compatible with 3D printing, various rheology modifiers have been employed to regulate the rheological characteristics of the inks. This is done to achieve the printability, shape retention and mechanical stability required for the final 3D printed structure. Modifiers such as functional polymers and inorganic nano-clays are utilized to enhance various 3D printing properties, which can improve the mechanical properties of printed hydrogels in addition to improving printability (Zhai et al., 2017).

#### 5.4.2 Multifunctional additives

To attain tailored properties for various applications, functional additives can be incorporated into the 3D printed hydrogel system, and the functional additives can make the hydrogel have better mechanical properties, better biocompatibility and targeted drug delivery capabilities. These additives encompass living cells, functional polymers, particles and ECM. ECM-mimicking hydrogels can promote cell attachment, proliferation, differentiation, and migration, effectively generating hydrogels that mimic the extracellular environment of native tissues can greatly enhance the viability and functionality of implanted tissue constructs (Gungor-Ozkerim et al., 2018). Kolesky et al. (2016) showcased the potential of bioprinting in generating vascularized thick tissues with high fidelity and biological relevance, and showed that the ability of printed hydrogels to support cell spreading and proliferation further highlighted their biological activity. They created functional tissues with multiple cell types, including stroma, parenchyma, and endothelium, that were able to support the transport of growth factors for over 6 weeks. This achievement highlights the ability of bioprinting technology emulating the intricate structure and function of native tissues. Moreover, it opens up exciting opportunities for regenerative medicine and tissue engineering, as it enables the precise control of cell placement and the construction of 3D architectures that can potentially integrate with host tissues.

## 6 Practical applications and clinical translation of 3D-printed hydrogels

Despite substantial progress, the clinical value of 3D-printed hydrogels depends on whether they can progress beyond laboratory-scale scaffold fabrication to reproducible, application-oriented products (Wang et al., 2023). In practice, one of the most mature directions is wound care, where 3D-printed hydrogel dressings can be customized to match irregular wound geometries and loaded with antibacterial agents, growth factors, or cells to improve moisture retention, drug delivery, and tissue regeneration (Zhou et al., 2024). Recent reviews have emphasized their particular promise for chronic and diabetic wounds, where patient-specific dressings and multifunctional bioactive formulations may offer advantages over conventional passive dressings (Zhou et al., 2024; Cui et al., 2025). Cartilage repair is another realistic application area, because hydrogels can be printed into anatomically defined, cell-supportive constructs that mimic the hydrated microenvironment of native cartilage; however, long-term mechanical durability, integration with host tissue, and restoration of zonal cartilage architecture remain major barriers (Doyle et al., 2021; Corrado et al., 2025). Similarly, in bone and osteochondral engineering, 3D-printed hydrogel systems are attractive as bioactive carriers or hybrid scaffolds, but their limited intrinsic stiffness usually requires reinforcement with ceramics, synthetic polymers, or composite structures (Lu J. et al., 2025; Turnbull et al., 2018; Lu K. et al., 2025). Overall, the most immediate translational opportunities appear to be in relatively accessible, non-load-bearing or moderately load-bearing applications such as wound dressings, cartilage patches, and localized defect fillers, rather than fully implantable large-volume organs (Zhou et al., 2024; Doyle et al., 2021; Turnbull et al., 2018).

From a translational standpoint, the major bottlenecks are not only biological but also manufacturing and regulatory (Wang et al., 2023; Singh et al., 2026). Cell-laden or multifunctional hydrogel constructs must be produced under sterile and highly controlled conditions, and scale-up requires consistent bioink formulation, stable rheological behavior, reproducible crosslinking, and quality control compatible with GMP-like workflows (Wang et al., 2023; Singh et al., 2026). In addition, clinical adoption will depend on matching degradation kinetics with tissue regeneration, ensuring vascularization in thick constructs, and generating preclinical evidence that printed hydrogels maintain functional performance after implantation rather than merely demonstrating *in vitro* printability (Doyle et al., 2021; Corrado et al., 2025; Singh et al., 2026). Therefore, future development should focus less on simply demonstrating printability and more on building clinically robust systems with standardized evaluation criteria, manufacturable processes, and clearly defined target indications (Corrado et al., 2025; Singh et al., 2026).

## 7 Future research perspectives

Despite the remarkable progress of hydrogel-based bioprinting, several important challenges must still be addressed before this technology can achieve broader clinical and industrial translation. One major issue is scalability and manufacturing reproducibility. Although many hydrogel systems perform well in laboratory-scale studies, the transition to large-scale and standardized production remains difficult because bioink formulation, rheological stability, crosslinking behavior, and post-printing consistency must all be tightly controlled (Wang et al., 2023; Singh et al., 2026). Future research should therefore focus on developing robust biofabrication workflows that are compatible with scale-up, automation, and quality control under clinically relevant manufacturing conditions (Wang et al., 2023; Singh et al., 2026).

Another critical challenge is vascularization, particularly for thick or complex tissue constructs. In many current studies, hydrogel-based printed scaffolds show promising *in vitro* printability and cytocompatibility, but their *in vivo* functionality is still limited by insufficient nutrient diffusion, oxygen transport, and vascular integration after implantation (Kolesky et al., 2016; Doyle et al., 2021; Corrado et al., 2025). This limitation is especially relevant for osteochondral and large-volume tissue constructs, where biological performance depends not only on structural fidelity but also on the formation of functional vascular networks (Kolesky et al., 2016; Doyle et al., 2021). Future work should therefore prioritize strategies such as multi-material bioprinting, gradient scaffold design, and the incorporation of angiogenic cues or vascular-supporting cell populations to improve long-term tissue survival and integration (Kolesky et al., 2016; Corrado et al., 2025).

In addition, mechanical performance and biological functionality must be more effectively balanced. Many printable hydrogels are designed primarily for extrusion fidelity or photopolymerization efficiency, but these properties do not always correlate with the mechanical robustness or biological functionality required for tissue regeneration (Wang et al., 2023; Doyle et al., 2021). For example, hydrogels used in bone and osteochondral engineering often require reinforcement with ceramics, nanomaterials, or polymeric secondary networks to achieve adequate load-bearing performance (Lu J. et al., 2025; Turnbull et al., 2018; Lu K. et al., 2025). Accordingly, future studies should move beyond simple printability assessments and focus on structure–property–function relationships that integrate printability, degradation kinetics, mechanical stability, and tissue-specific cellular responses (Doyle et al., 2021; Turnbull et al., 2018; Lu K. et al., 2025).

A further issue is the lack of regulatory and translational standardization. For clinical application, hydrogel-based bioprinted constructs must meet strict requirements related to sterility, reproducibility, storage, preclinical validation, and regulatory classification, especially when they contain living cells or multifunctional bioactive components (Wang et al., 2023; Singh et al., 2026). At present, these regulatory considerations are often discussed only after proof-of-concept studies, rather than being integrated into material and process design from the beginning (Singh et al., 2026). Future research should therefore incorporate translational criteria earlier in development, including standardized testing protocols, reproducible manufacturing strategies, and clearly defined therapeutic indications (Wang et al., 2023; Singh et al., 2026).

Overall, the next stage of hydrogel-based bioprinting should shift from demonstrating technical feasibility toward establishing clinically robust, scalable, and functionally validated platforms. Progress in this field will depend not only on the development of advanced bioinks, but also on closer integration of material science, biofabrication engineering, vascular biology, and regulatory planning. Through such interdisciplinary efforts, hydrogel-based bioprinting may move more effectively from proof-of-concept research toward practical biomedical applications (Wang et al., 2023; Doyle et al., 2021; Singh et al., 2026).

## 8 Conclusion

Three-dimensional printing of hydrogels has become an important strategy in tissue engineering because it enables the fabrication of scaffolds with controllable architecture, tunable properties, and biomimetic microenvironments. However, no single printing technique or hydrogel system is universally suitable for all applications. Instead, the choice of printing method should be based on the balance among resolution, printability, cytocompatibility, mechanical stability, and the biological requirements of the target tissue. In general, light-based techniques provide higher structural precision, whereas extrusion-based approaches offer broader material compatibility and are better suited for cell-laden bioinks. Similarly, natural hydrogels are advantageous for their bioactivity and resemblance to the extracellular matrix, while synthetic hydrogels provide better control over composition and mechanical performance. Recent progress in composite, tough, and multifunctional hydrogels has further expanded the potential of 3D printing in bone, cartilage, vascular, and soft tissue engineering. Nevertheless, several challenges remain, including insufficient long-term mechanical stability, limited vascularization, difficulties in integrating biological activity with printability, and barriers to large-scale and clinically translatable fabrication. Future development should therefore focus not only on novel printable hydrogel systems, but also on better integration of material design, printing strategy, and tissue-specific functional demands. Overall, the next stage of hydrogel-based 3D printing should move beyond proof-of-concept scaffold fabrication toward the development of more standardized, functionally robust, and clinically translatable tissue-engineering platforms.
